# Lipid Content and Composition of Oocytes from Five Coral Species: Potential Implications for Future Cryopreservation Efforts

**DOI:** 10.1371/journal.pone.0057823

**Published:** 2013-02-28

**Authors:** Chiahsin Lin, Li-Hsueh Wang, Pei-Jie Meng, Chii-Shiarng Chen, Sujune Tsai

**Affiliations:** 1 Department of Biotechnology, Mingdao University, Peetow, Chang Hua, Taiwan; 2 National Museum of Marine Biology & Aquarium, Checheng, Pingtung, Taiwan; 3 Institute of Marine Biotechnology, National Dong Hwa University, Checheng, Pingtung, Taiwan; University of Central Florida, United States of America

## Abstract

Given the previously documented importance of lipid concentration and composition in the successful cryopreservation of gorgonian corals, these parameters were assessed in oocytes of five species of scleractinian coral; *Platygyra daedalea*, *Echinopora gemmacea*, *Echinophyllia aspera*, *Oxypora lacera* and *Astreopora expansa*. Wax esters, phosphatidylethanolamine, phosphatidylcholine, and fatty acids were all measured at detectable levels, and the latter were produced at significantly elevated quantities in *E. gemmacea*, *E. aspera,* and *O. lacera*. On the other hand, phosphatidylethanolamine, phosphatidylcholine, and wax ester were found at significantly higher concentrations in *A. expansa* oocytes. Triacylglycerol was not present in any species. Interestingly, the total lipid content of oocytes from all five scleractinians was significantly lower than that of oocytes of two gorgonian species, *Junceella juncea* and *Junceella fragilis*. As higher total lipid concentrations may be correlated with greater degrees of cellular membrane fluidity at lower temperatures, it stands to reason that gorgonian coral oocytes may be more likely to survive the cryopreservation process than oocytes of scleractinian corals.

## Introduction

As numerous coral species across the globe are threatened by extinction due to phenomena such as global climate change [1], [2], cryopreservation of coral germ cells has recently been attempted as a potential *ex situ* conservation technique for coral population preservation. In particular, l*ow temperature* preservation of coral oocytes has become an essential tool to conservation biologists, as the negative effects of the cryopreservation process (i.e., cryo-injuries due to low temperature sensitivity) are not as evident in this early life history stage as they are in adults [3]. Previous studies [3], [4] have indicated that the scleractinian coral *Echinopora* sp. and the gorgonian**s** *Junceella juncea* and *Junceella fragilis* demonstrate significant degrees of cooling tolerance at 5 and 0°C, but their oocytes do not demonstrate such cryosensitivity until the temperature is reduced to −5°C. Specifically, ATP levels decreased dramatically after four hours of chilling at the latter temperature in these oocytes [3].

Although all macromolecules are subjected to disruption or degradation due to ice crystal formation inherent to the cryopreservation process, cell membranes are known to be particularly sensitive [5] and may serve as the “weakest link” in ultimately determining the success of cryopreservation. As such, an understanding of coral phospholipid and other lipid content may prove fruitful in ultimately gauging the ability to cryopreserve corals. Prior work has investigated the importance of lipids in coral biology [6]–[9], and, specifically, lipids have been shown to be important energy sources for growth [6] and reproduction [10]. In some corals, lipids can comprise 10–40% of the tissue biomass [6]–[9], explaining why energy-deprived corals can survive up to 114 days without their endosymbiotic dinoflagellates [11]. Immense lipid concentrations have also been documented in coral eggs, in which up to 80% of the volume can be occupied by lipid droplets [12].

Studies have suggested that sensitivity of coral oocytes to low temperature may be dependent upon intracellular lipid content and/or composition [3], [4]. While little is known about the importance of intracellular lipid content in determining the cryosensitivity of coral oocytes, it is possible that a positive relationship exists between these two parameters given the role of lipids in membrane fluidity. As such, the role of lipid composition in driving changes in membrane fluidity could provide one cellular mechanism by which these corals could adapt to exposure to low temperature [13]. In order to ultimately establish such a relationship between lipid concentration/composition and cryosensitivity, the present study documented not only the concentration of total lipids, but also the concentrations of neutral lipids (wax ester and triacylglycerol), fatty acids, and polar lipids (phosphatidylethanolamine and phosphatidylcholine) in oocytes from five species of scleractinian coral; *Platygyra daedalea*, *Echinopora gemmacea*, *Echinophyllia aspera*, *Oxypora lacera*, and *Astreopora expansa*.

## Results

### Lipid Composition of Oocytes from Five Species of Scleractinian Coral

**Figure 1:**
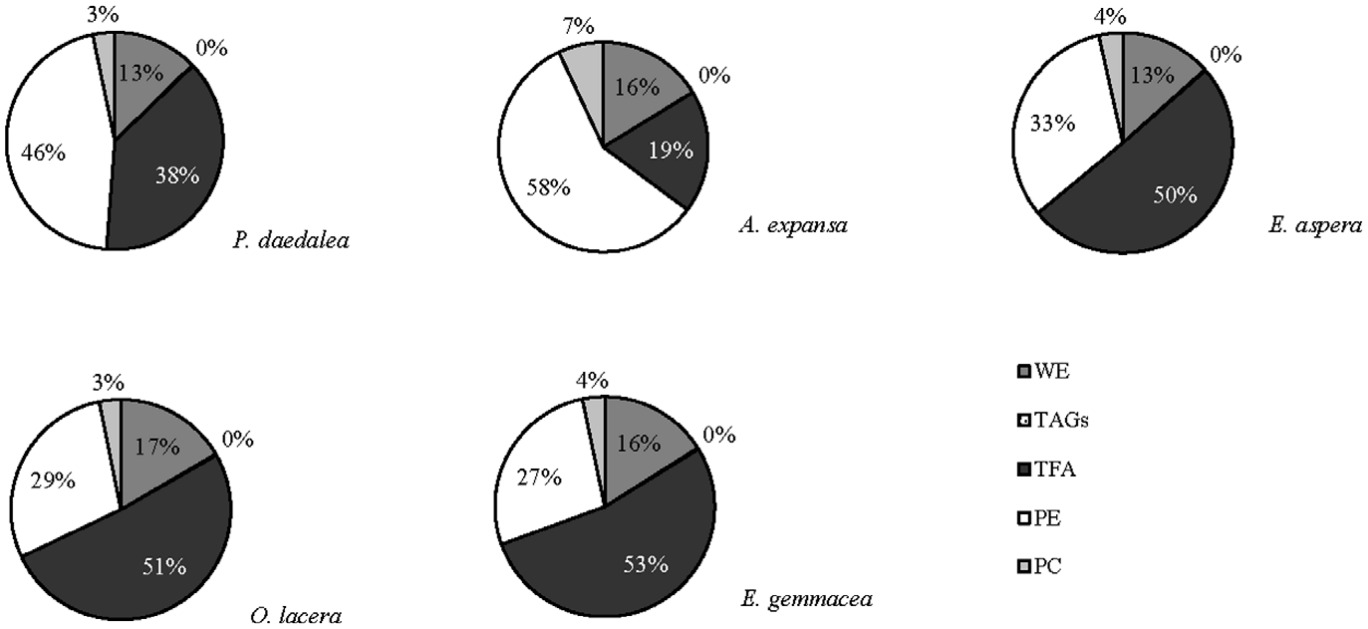
Relative concentrations of wax esters (WE), triacylglycerol (TAGs), fatty acids (FA), phosphatidyethanolamine (PE), and phosphatidylcholine (PC) of oocytes from five coral species.

Oocytes of each of the five target scleractinian coral species were an average size of 0.05±0.02 mm^3^. The concentrations (expressed as percentages of the total lipid concentration) of the individual lipid classes are shown in Fig. 1. The total lipid composition was similar across species, and the main oocyte lipid species were wax esters (WE), fatty acids (FA), phosphatidylethanolamines [PE], and phosphatidylcholines [PC]. The dominant lipid constituents in *E. gemmacea*, *E. aspera* and *O. lacera* oocytes were fatty acids (53%, 50%, and 51%, respectively). FA concentrations were lower in *P. daedalea* (38%) and *A. expansa* (19%) (Fig. 1). Higher concentrations of PE were found in *P. daedalea* and *A. expansa* (46% and 58%, respectively) than *E. gemmacea*, *E. aspera*, and *O. lacera* (27, 33, and 29%, respectively). The relative concentrations of WE and PE were 3–13% and 7–17%, respectively, for all species (Fig. 1). There was no triacylglycerol (TAG) detected any of five species (Fig. 1).

### Interspecific Differences in Coral Oocyte Lipid Composition

**Figure 2:**
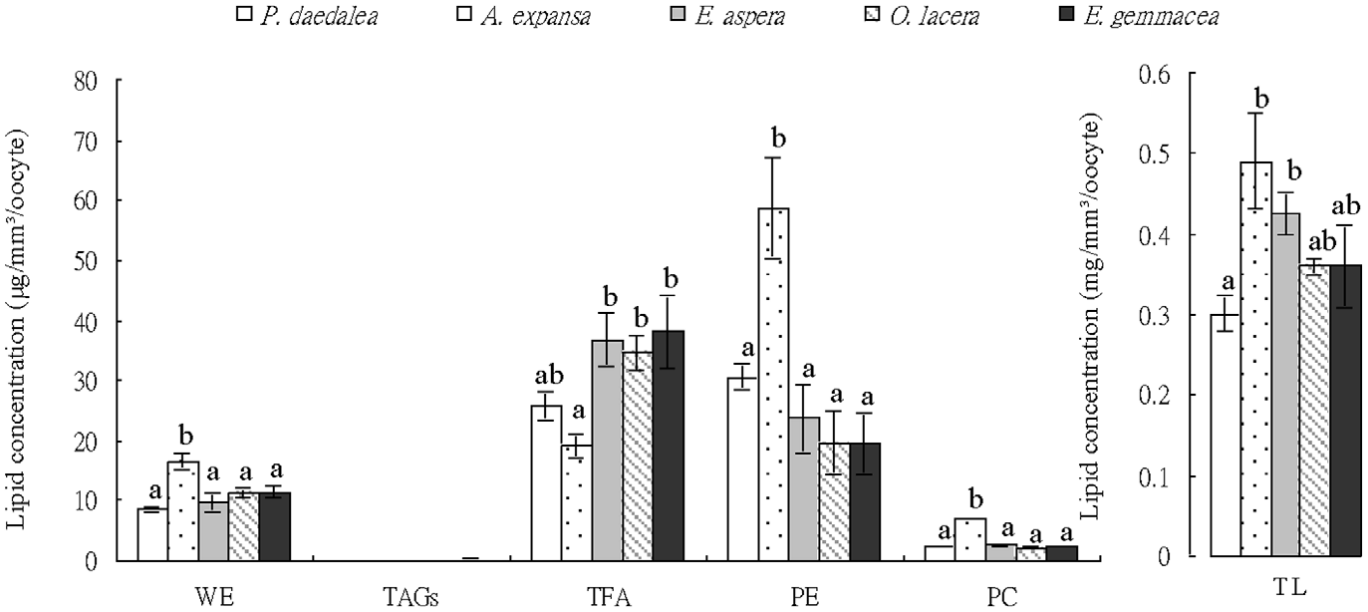
Concentrations of total lipids (TL), wax esters (WE), fatty acids (FA), phosphatidyethanolamine (PE), and phosphatidylcholine (PC) extracted from oocytes of *P. daedalea*, *E. gemmacea*, *E. aspera*, *O. lacera*, and *A. expansa*. Error bars indicate standard *error* of the mean. Statistically significant differences (Tukey’s HSD, *p<0.05*) within each lipid species are denoted by letter groups.

The total lipid concentration of oocytes from *P. daedalea* (0.30±0.02 mg/mm^3^/oocyte) was significantly lower (Tukey’s HSD, *p>0.05*) than those of oocytes of *A. expansa* and *E. aspera* (0.49±0.06 and 0.42±0.03 mg/mm^3^/oocyte, respectively), whilst the total lipid concentration of *E. gemmacea* (0.36±0.05 mg/mm^3^/oocyte) did not differ significantly from any of the other species (Fig. 2). The concentration of FA (Fig. 2) was significantly higher (*p>0.05*) in *E. gemmacea*, *E. aspera*, and *O. lacera* oocytes (38.14±6.10, 36.72±4.52 and 34.62±2.89 µg/mm^3^/oocyte, respectively) compared to those of *A. expansa* (19.19±1.90 µg/mm^3^/oocyte). The absolute concentrations of PE and PC were significantly higher (*p>0.05*) in *A. expansa* (58.70±8.50 and 7.12±0.06 µg/mm^3^/oocyte, respectively) than in oocytes of the other species, whose PE and PC concentrations ranged from 19.54±5.28 to 30.59±2.03 µg/mm^3^/oocyte and from 2.14±0.02 to 2.56±0.05 µg/mm^3^/oocyte, respectively. PE concentration did not differ significantly between oocytes of *P. daedalea*, *E. gemmacea*, *E. aspera*, and *O. lacera* (Fig. 2). On the other hand, the concentration of WE was significantly higher in oocytes of *A. expansa* (16.50±1.40 µg/mm^3^/oocyte) compared with those of the other species, whose concentrations ranged from 8.53±0.49 to 11.50±0.90 µg/mm^3^/oocyte (Fig. 2).

## Discussion

Most shallow water corals have been shown to possess high concentrations of WE and TAG, which collectively account for 40–73% of their total lipid content [6], [9], [14]. Conversely, another study with the shallow water species *Montipora digitata* found that decreased concentrations of WE and TAG were associated with an increased degree of energy expenditure by proliferating cells [15], potentially implicating that these macromolecules are important energy storage reservoirs. Collectively, the results of these prior studies suggests that WE and TAG are the most important lipid species with respect to energy storage, and their concentrations typically ranged from 13–17%. Interestingly, while the WE concentrations observed herein were in line with those of these prior studies (i.e., 13–17%), TAG was detected in only very small quantities (<1%) in oocytes of the five species. Such a low standing concentration of this lipid relative to the others may suggest that it is amongst the first to be metabolized, while WE are used predominantly as energy storage reserves. Therefore, in coral oocytes, it appears that WE is relatively more important in terms of its capacity to serve as an energy storage compound compared to TAG.

Few articles have been dedicated to the FA composition of reef-building corals, though studies do exist for *M. digitata* [15], as well as *Gorgonia mariae* and *Gorgonia ventalina* [16]. Montiporid corals typically contain 102–103 *Symbiodinium* sp. in an egg at the time of spawning [17], and these photosynthetically active dinoflagellate undergo division during embryogenesis of the coral host. Ultimately, these endosymbiotic microbes are important in coral development and health, as they readily translocate carbon compounds to the host [18]. In gorgonians, lipid levels are dominated by a high proportion of FA in maturing oocytes [13]. Other studies have also shown that corals contain high levels of unsaturated FA from plankton capture, whilst corals containing a greater amount of saturated FA rely more on the translocation of photosynthetic products from their *Symbiodinium* populations [19]. In the present study, *P. daedalea*, *E. gemmacea*, *E. aspera*, and *O. lacera* oocytes, which all inherit *Symbiodinium* from their parents, contained more FA than *A. expansa* oocytes, which are azooxanthellate. It is possible, then, that the FA concentrations were influenced by the presence of dinoflagellate endosymbionts. For some reef-building coral species, high phospholipase activity could lead to such enrichment of FA levels [20]. Related studies are currently proceeding in our laboratory.

In a previous study with the soft coral *Gersemia rubiformis* [20], it was found that the majority of the polar lipids (>80%) were phospholipids, and the main phospholipids were PE, PC, and phosphatidylserine [20]. Similarly, two species sampled in Hawaii had high levels of PE and PC in the polar lipid fraction [21]. A similar phospholipid distribution was observed in three species of tropical gorgonians (*Psammogorgia nodosa*, *Bebryce indica*, and *Mopsella aurantia*), in which PE and PC comprised 40% and 30%, respectively, of the total lipid fraction [22]. Our previous study [21] indicated that two gorgonians, *J. juncea* and *J. fragilis*, produced more PC than PC, and similar results were observed herein. Although the concentrations of PE and PC were 42.46 µg/mm^3^/oocyte and 96.32 µg/mm^3^/oocyte and 10.02 µg/mm^3^/oocyte and 10.17 µg/mm^3^/oocyte in *J. juncea* and *J. fragilia*, respectively [13], in the present study, the concentrations of PE and PC were notably lower, ranging from 19.54 to 58.70 µg/mm/oocyte and 2.15 to 7.12 µg/mm/oocyte, respectively. As a result, PE is likely to be the more abundant phospholipid species in the cellular membranes. PE and PC create a higher surface viscosity and result in fluid lipid membranes with lower melting points [23]. The higher levels of PE and PC in the two gorgonian oocytes may be due to their inhabitance of greater depths, and hence greater pressures and lower temperatures, relative to shallow water corals, such as those sampled herein; in fact, both abiotic influences could necessitate adjustments in cellular membrane fluidity [13].

Oocytes of the five species of scleractinian coral sampled herein, which typically live at 3–5 m depth, had similar overall lipid concentrations, as well as a similar distribution of lipids across the four lipid species detected. Previous studies have found that scleractinian coral (*Echinopora* spp.) oocytes were very sensitive to chilling, and their high intracellular lipid levels may serve as a possible link to their cryosensitivity [3]. The fact that the total lipid content, was significantly lower in the oocytes of the five species of scleractinian coral relative to the gorgonian oocytes may suggest that the latter, deeper-water species may possess such higher lipid levels in order to increase membrane fluidity. If such an increase in membrane fluidity is indeed characteristic of these gorgonians, they may produce oocytes that are better candidates for cryopreservation research, as it is generally thought that species with greater degrees of cell membrane fluidity will more likely survive the cryopreservation process.

## Materials and Methods

### Collection of Scleractinian Coral Oocytes

**Figure 3:**
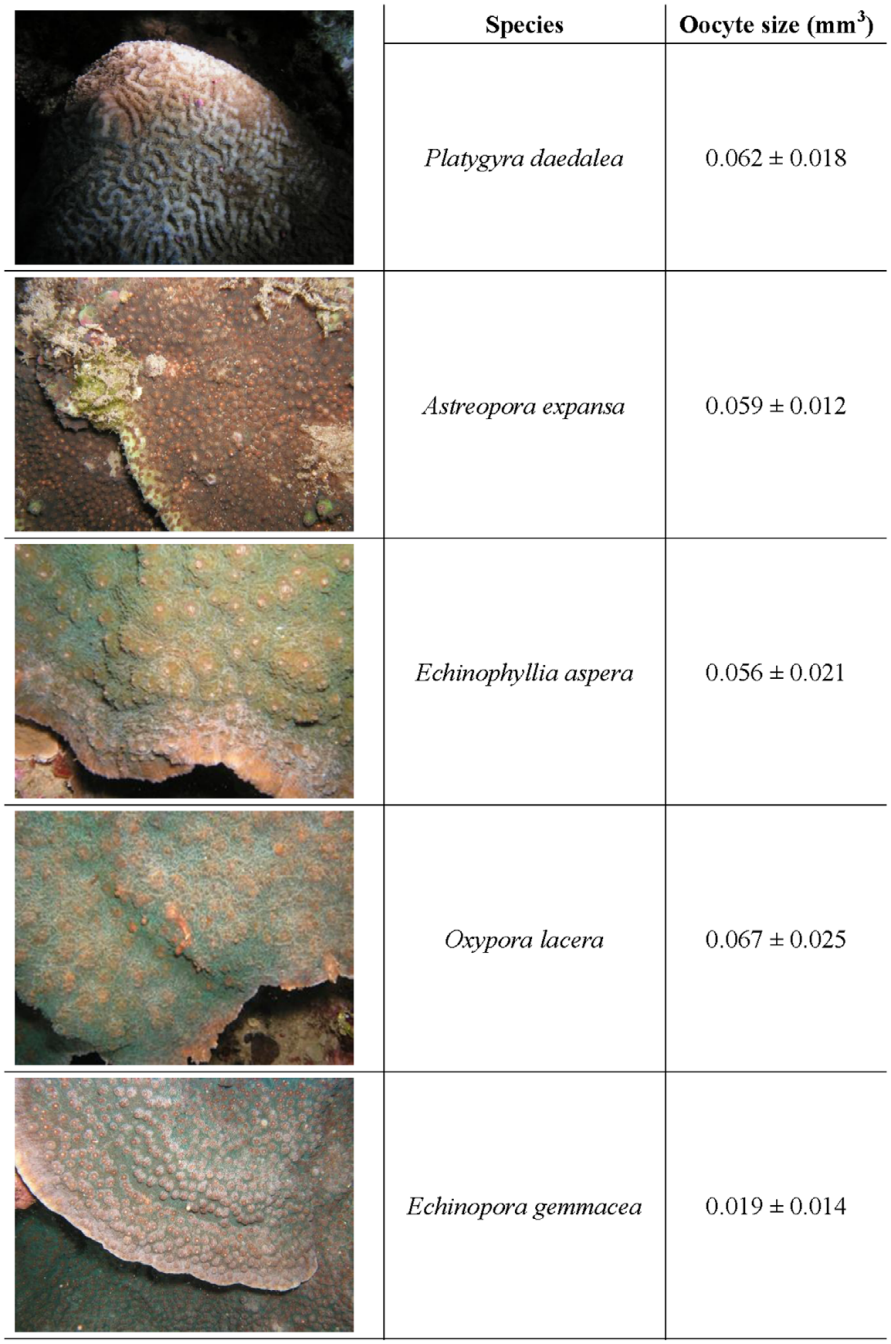
Representative images of the five target coral species used in the study. Their respective oocyte sizes (± standard error of the mean) are displayed in the adjacent column.

In early 2011, SCUBA divers randomly selected one representative colony of each of five species of scleractinian coral; *P. daedalea*, *E. gemmacea*, *E. aspera*, *O. lacera* and *A. expansa* (Fig. 3), at a depth of 3 to 5 m in Nanwan Bay, Taiwan (21°56′N, 120°44′E). Between April and May 2011, oocytes were collected *in situ* at night by SCUBA divers following the method developed by [4] *during* coral *spawning events.* Oocytes were immediately transported back to the laboratory and kept in an aquarium containing filtered (0.45 µm) natural seawater at 25°C for further processing. The coral collection was approved by the Kenting National Park Management Office.

### Examination of Coral Species

In order to determine the identity of the sample corals to species level, polyp and sclerite morphology were analyzed under a light microscope (C31, Olympus, Japan), and pertinent features (e.g., corallite structure) were used with a key [24]. Additionally, samples were preserved in fixation buffer (30% sodium hypochlorite solution, Sigma, USA) for 10 hours and washed with distilled water before drying. The samples were then sent to another laboratory (S Horng, Taiwan Association for Marine Environmental Education, Taipei) for independent verification. In all cases, the two laboratories came to the same conclusions regarding species level identification of the five target corals.

### Lipid Analysis

Three pools of 50 oocytes from each species were analyzed. Triplicate technical replicates were then employed to analyze the lipid content in each of the three pools from each species. We therefore obtained a total of nine measurements from a total of 150 oocytes from each species. The oocytes were immersed in a solution of 4 ml dichloromethane and 2 ml methanol, and lipids were extracted as in [22]. The extracted lipids were first normalized. Then, high performance liquid chromatography (HPLC) with an evaporative light scattering detector (ELSD) was used to distinguish the lipid species [16]. The HPLC-ELSD system was comprised of a Hitachi Model L7100 HPLC pump connected to a Sedex 80 evaporative light-scattering detector (Sedex, France) with an auto-sampler (Hitachi, L7200, Japan). Separations were performed with an YMC-PVA-SIL column (100 × 3 mm i.d.; 5 mm particles; Hichrom Ltd, UK), and nitrogen gas was used to evaporate the solvent. The ELSD drift tube had a nebulizer gas flow rate of 2.5 kg/cm^2^ with nebulization temperatures set at 55°C in the ELSD drift tube (Table 1).

**Table 1:** Gradient elution program for HPLC-ELSD-based separation.

Time (min)		0	4	5	10	12	15	18	20	25	30	40
	A(%)	100	100	85	80	75	50	30	30	25	30	100
Solvents	B(%)	0	0	15	20	25	50	50	40	30	70	0
	C(%)	0	0	0	0	0	0	20	30	45	0	0
Flow rate (ml/min)		1.0	1.0	1.0	1.0	1.0	1.0	1.0	1.0	1.0	1.0	1.0

### Statistical Analysis

*Each HPLC analysis was repeated three times for each of the three pseudo-replicates from each coral colony*. One-way ANOVA was used to examine the effect of coral species on lipid composition after verifying data normality (Kolmogorov-Smirnov test) and homoscedasticity (Levene’s test). Tukey’s post-hoc tests were used to determine if there were significant differences between individual means with SPSS software (Version 17.0; SPSS Inc., Chicago, IL, USA). In all statistical tests, *p* values less than 0.05 were considered to be significant. All results are presented as mean ± SEM.
